# Responses of nutrient capture and fine root morphology of subalpine coniferous tree *Picea asperata* to nutrient heterogeneity and competition

**DOI:** 10.1371/journal.pone.0187496

**Published:** 2017-11-02

**Authors:** Dandan Li, Hongwei Nan, Jin Liang, Xinying Cheng, ChunZhang Zhao, HuaJun Yin, ChunYing Yin, Qing Liu

**Affiliations:** 1 Key Laboratory of Mountain Ecological Restoration and Bioresource Utilization, Chengdu Institute of Biology, Chinese Academy of Sciences, Chengdu, China; 2 Ecological Restoration Biodiversity Conservation Key Laboratory of Sichuan Province, Chengdu Institute of Biology, Chinese Academy of Sciences, Chengdu, China; 3 College of Forestry, Shanxi Agricultural University, Jinzhong, China; Pacific Northwest National Laboratory, UNITED STATES

## Abstract

Investigating the responses of trees to the heterogeneous distribution of nutrients in soil and simultaneous presence of neighboring roots could strengthen the understanding of an influential mechanism on tree growth and provide a scientific basis for forest management. Here, we conducted two split-pot experiments to investigate the effects of nutrient heterogeneity and intraspecific competition on the fine root morphology and nutrient capture of *Picea asperata*. The results showed that *P*. *asperata* efficiently captured nutrients by increasing the specific root length (SRL) and specific root area (SRA) of first-and second-order roots and decreasing the tissue density of first-order roots to avoid competition for resources and space with neighboring roots. The nutrient heterogeneity and addition of fertilization did not affect the fine root morphology, but enhanced the P and K concentrations in the fine roots in the absence of a competitor. On the interaction between nutrient heterogeneity and competition, competition decreased the SRL and SRA but enhanced the capture of K under heterogeneous soil compared with under homogeneous soil. Additionally, the P concentration, but not the K concentration, was linearly correlated to root morphology in heterogeneous soil, even when competition was present. The results suggested that root morphological features were only stimulated when the soil nutrients were insufficient for plant growth and the nutrients accumulations by root were mainly affected by the soil nutrients more than the root morphology.

## Introduction

Nutrients are normally heterogeneously distributed in soil [1–4]. The root system is the major plant organ for acquiring nutrients and water [5] and is able to forage for resources. Roots can identify nutrient hotspots and exhibit morphological or physiological responses to nutrient heterogeneity [6–8]. Studies have shown that there are positive, negative and neutral effects of nutrient heterogeneity on plants, depending on the species [9–12]. In addition, some studies have also shown that plant responses to nutrient heterogeneity are associated with neighboring plants [11,13,14]. Nutrition competition from neighboring plants limits plant growth by regulating nutrient bioavailability. From an ecological perspective, competition among plants influences the structure and function of ecosystems. Therefore, researchers have begun to study the effect of competition from neighboring plants on roots, including root morphology and growth [1,11,15,16].

Nutrient heterogeneity and competition from neighbors are two important factors that affect plant growth [6,8,11,13,15]. Plants are simultaneously exposed to nutrient heterogeneity and competition from neighboring plants in the natural environment. Increasing attention has been focused on studying the effects of interactions between these two factors [11–13,17]. There are contradictory conclusions regarding the relationships between resource heterogeneity and intensity of competition on plants. Newman [16] and Tilman [18] have reported that competition intensity does not vary with resource variability or productivity. However, Hutchings et al. [19] found that the localization of nutrients within patches under heterogeneous conditions increased the intensity of competition due to higher productivity and higher resource demands. In addition, Janecek et al. [13] showed that selective root placement in a nutrient patch was not affected by interspecific competition. To date, most information on the mechanisms of below-ground competition or nutrient heterogeneity has been collected from studies of herbaceous and scrub vegetation [9,11–12,20,21], whereas knowledge of responses to belowground competition or nutrient heterogeneity in forest ecosystems is limited. It is necessary to simultaneously investigate the responses of trees to nutrient heterogeneity and competition in forest ecosystems to provide a greater understanding of those influences on tree growth and forest ecosystems.

Water and nutrient uptake mainly occur in fine roots with a diameter of ≤2 mm [22]. Fine roots have numerous branching orders [22], which markedly differ in form and function [20]. For example, distal roots are categorized as first-order, roots from which two first-order roots branched are categorized as second-order, and so on. Roots of different branch orders play various roles in below-ground nutrient cycling, with higher orders (i.e., ≥ third order) predominantly used for transport, storage and structural support and lower orders (i.e., first and second) mainly used for the acquisition of nutrients and water [23]. The morphological characteristics of roots (including the root tissue density, diameter, specific root length (SRL) and the specific root surface area (SRA)) vary with the branch order and are associated with nutrient accumulation. Although a few studies have used root morphology to demonstrate responses to competition [24,25], no study has yet described the responses of branch order-dependent root morphology on the interactions between nutrient heterogeneity and competition. In addition, most studies only use plant growth measures, such as biomass, to determine the effects of interactions between nutrient heterogeneity and competition on plants [6,11,13,21,24,25]. If we can supply additional data concerning variations in plant morphology with branch order roots to measure those effects on plants, it would improve the understanding of the response of plants to nutrient heterogeneity and competition.

The accumulation and distribution of the elements such as N, K and P in plants is a primary determinant for plant growth [26]. Studying the accumulation and distribution of these mineral elements is important for forest management. There are a number of studies that show that the uptake of N decreases with increasing root order [27–29]. Pregitzer et al. [30] further demonstrated that nitrogen accumulation was related to the fine root morphology, such as the root length, specific root length and specific root area. However, these studies did not consider the effects of nutrient heterogeneity and/or competition when they studied the relationship between nutrient uptake and root morphology. In a previous work by our team, Nan et al. [31] focused on the responses of the root foraging ability to nutrient heterogeneity and competition, but only investigated the root architecture of spruce seedlings as indicators of the root foraging ability and did not directly investigate nutrient uptake in roots. This study did not clearly explain the real influential mechanism of nutrient heterogeneity or competition because they did not investigate the single effect of nutrient heterogeneity and competition, respectively. Furthermore, although both the root architecture and root morphology could affect the root foraging ability, they may show different responses when plants are subjected to nutrient heterogeneity and competition. Thus, further study on the effects of competition or nutrient heterogeneity as well as their combined effects on root morphology and nutrient uptake is required.

In the present study, we selected seedlings of spruce (*Picea asperata*) because spruce is widely distributed in the subalpine coniferous forests of western Sichuan, China. *Picea asperata* is one of the dominant tree species in plantations of this region and has a significant ecological influence on subalpine forests. In addition, subalpine coniferous forests constitute the second largest biome in China [32]. Studying the response of spruce to the combined effect of competition from neighbors and nutrient heterogeneity in soils will provide a scientific basis for the management of forests. Thus, we conducted two split-pot experiments to investigate the effects of the nutrient heterogeneity in soil and intraspecific competition on the fine root morphology and uptake of K and P by fine roots that vary by branch order in *P*. *asperata*. We tested the following hypotheses: 1) the fine root morphology and nutrient accumulation will be restrained by competition for resources and space with neighboring plants; 2) the fine root morphology will be affected by nutrient heterogeneity, such as increased SRL, SRA in rich-nutrient pots and decreased SRL, SRA in poor-nutrient pots; 3) when the effects of competition and nutrient heterogeneity are combined, the competition intensity will depend on the nutrient distribution in the pot; and 4) the responses of nutrient accumulation to competition from neighbors and nutrient heterogeneity are associated with root morphology.

## Materials and methods

### Ethics statement

The experiment was conducted in an open field (31°25’N, 103°12’E, 2,309 m, a.s.l) at the Miyaoluo Natural Reserve of Lixian County, eastern Tibetan Plateau, Sichuan Province, China, with mean annual temperature, precipitation and evaporation 11.3°C, 764.4 mm, and 1450 mm, respectively. We obtained the permissions from the Forestry Bureau of Lixian County, and the forestry workers for the filed study. And we confirmed that our studies did not involve endangered or protected species. In addition, no specific permission was required for these locations because our study was the general pot experiment.

### Experimental design and treatments

**Fig 1:**
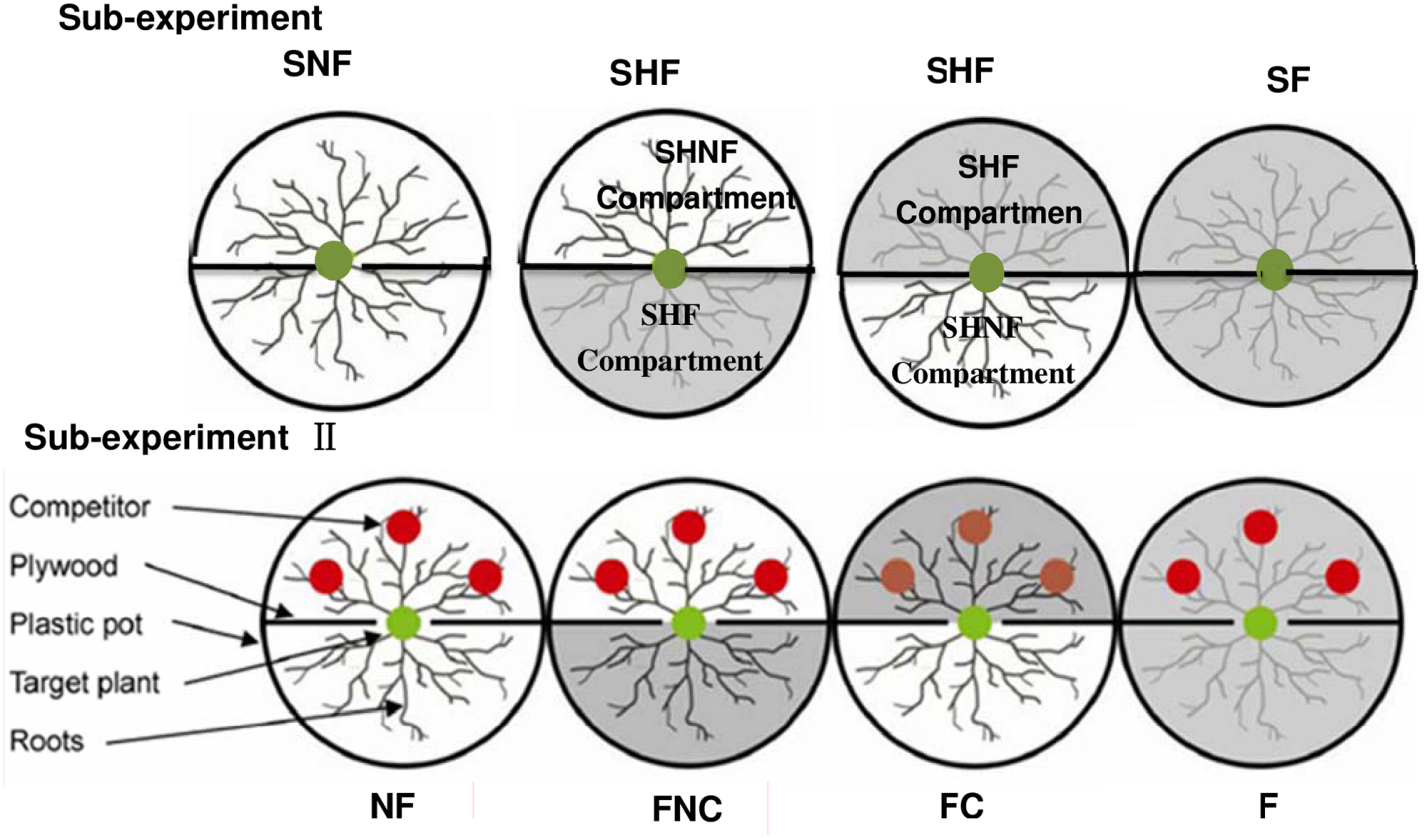
Schematic of the experimental treatments. The sub-experiment I included the upper three treatments without competitors with applying fertilizer to one half compartment (SHF) of the container or both two compartments (SF) of the container, or no fertilizer in both compartments (SNF) of the container. SHNF is the non-fertilizer compartment in SHF treatment and SHF is the fertilizer compartment of the same container in SHF treatment. The sub-experimentIIincluded the lower four treatments with competitors roots which was set up by applying fertilizer to the competitive compartment (with competitors) of the container (FC), the non-competitive compartment (without competitors) of the container (FNC), and both the two compartments of the container (F), as well as no fertilizer being in both two compartments of the container (NF). The gray parts represent applying fertilizer. The figure has been modified from previous work by our team [31].

We set up this experiment based on our previous research, Nan et al. [31]. The experiment included two sub-experiments (sub-experiment I without a competitor and sub-experiment with a competitor) (Fig 1). Sub-experiment I without a competitor was mainly used to investigate the influence of the soil nutrient distribution and fertilization on roots, and the sub-experimentIIwith a competitor was mainly used to investigate the effects of competition and its interaction with nutrient heterogeneity and fertilization on roots.

In sub-experiment I without competitors, only the targeted plant was used in the pot without any neighboring plants, and an approach comprising three treatments was adopted by applying fertilizer to one compartment (SHF) or both compartments of the pot (SF). In the SHF treatment, the compartment with fertilizer was called SHF and the other compartment of the same pot without fertilizer was called SHNF. Meanwhile, the control was set up by applying no fertilization to either compartment of the container (SNF) (Fig 1). Each treatment had eight replicates. First, a cylindrical pot (38 cm in diameter and 30 cm deep) was divided into two equal compartments with solid plywood planks. Three-year-old seedlings of *P*. *asperata* with similar sizes were randomly established in the pot. The root systems of these seedlings had nearly homogeneous and symmetrical distributions around the stem axis. One spruce seedling that was selected for use as the targeted plant was carefully placed in the middle of each pot. The main root of this seedling was then inserted into a narrow (3-cm) gap carved into the plywood plank, whereas the lateral roots were equally arrayed into separate compartments. The fertilizer contained N:P:K at a 15:1:1 ratio based on Hoagland’s hydroponic solution [33]. Fertilizer was applied from June to mid-September 2013 at 1.0 g N m^−2^ every 10 days (a total of 10 times throughout the growing season), the seedlings were watered as frequently as needed.

Corresponding to sub-experiment I, another approach comprising four treatments was adopted in sub-experimentIIwith a competitor. In this approach, three spruce seedlings were planted as competitors in one compartment of each pot (the competitive compartment), whereas the other compartment (non-competitive compartment) contained no seedlings (Fig 1). The treatments included fertilizer application either in the competitive compartment (with competitors) of the pot (FC), non-competitive compartment (without competitors) of the pot (FNC), or both compartments of the pot (F), as well as no fertilizer in both compartments of the pot (NF). Each treatment had eight replicates.

The pots were filled with sieved (4.5-mm mesh) and root-free soil collected from a nearby forest. To test the soil properties, the soil was finely ground (<60 um) and digested in concentrated nitric acid and hydrogen dioxide (HNO_3_-H_2_O_2_) for the determination of total P and K, Ca, Mg, Cu, Zn, Fe and Mn using inductively coupled plasma atomic emission spectrometry (ICP-AES) (Thermo Jarrell Ash, USA). A subsample was used to measure the total N using a CHN-elemental Analyzer (Vario MACRO, Elementar Analysesyteme GmbH, Hanau, Germany). The soil pH was measured by a pH meter at a ratio of 1:2.5 soil:water. The organic C was analyzed by a TOC analyzer (Multi-N/C 2100; Analytic Jean, Germany). The soil properties were as follows: pH: 5.85, organic C: 62.70 mg g^−1^, total N: 3.66 mg g^−1^, P: 0.43 mg g^−1^, K: 7.92 mg g^−1^, Ca: 7.9 mg g^−1^, Mg: 5.19 mg g^−1^, Cu: 0.035 mg g^−1^, Zn: 0.76 mg g^−1^, Fe: 27.7 mg g^−1^ and Mn: 0.454 mg g^−1^.

### Harvest and root morphological measurements

During mid-September 2013, all targeted plant seedlings were carefully harvested by hand while taking care to maintain the integrity of their root systems. Roots were then separated from each seedling and divided into two groups based on the compartment in which they were grown. All of the root systems in each group were carefully washed free of soil. To obtain accurate estimates of the morphological variables, the washed root networks were strictly isolated by branch order [34]. For example, the distal roots were categorized as first-order, and the roots from which two first-order roots branched were categorized as second-order. In the present study, the identified fourth- and fifth-order roots were combined as fourth-order roots. Roots of different orders were scanned. The diameters of all five orders of roots were <2 mm. Because roots of lower branch orders are more sensitive to environmental changes than those of higher branch orders, we took the responses of the first three root orders of root morphology into account to evaluate competition and nutrient heterogeneity. The root morphological variables, including the root length, root diameter and root area, as well as the individual biomass values of different orders, were measured using the WinRHIZO image analysis software (Regent instruments, Quebec, QC, Canada). SRL (cm g^−1^), SRA (cm^2^ g^−1^) and tissue density (g cm^−3^) were also calculated. SRL denoted the fine root length per unit mass of the fine root, SRA denoted the fine root surface area per unit mass of the fine root, and tissue density denoted the dry weight per unit volume of the fine roots.

### Nutrient analyses

The shoots of the target trees were separated from the roots and divided into three parts: the stems, branches and leaves. The roots and the shoots were finely ground (<100 um). Plant sample of 0.5g were weighed and digested in concentrated nitric acid and hydrogen dioxide (HNO_3_-H_2_O_2_). After that, the digested solution was transferred into a 10 ml volumetric flask and diluted into 10 ml with distilled water. Finally, the concentrations of K and P in final solution were determined by inductively coupled plasma atomic emission spectrometry (ICP-AES) (Thermo Jarrell Ash, USA). The concentrations of K and P in roots of different orders and the shoots of different parts were calculated by their concentrations in the solutions.

### Statistical analyses

To assess root responses, we analyzed the root morphology and nutrient concentration in root over the two compartments of each pot. We used ANOVA to test the effects on the root morphology and root nutrient concentration with competition (with competitor *vs*. without competitor), nutrient heterogeneity (homogeneous *vs*. heterogeneous), fertilization (no fertilizer *vs*. fertilizer) and compartment (competitive *vs*. non-competitive compartment) as fixed factors and the root morphology and root nutrient concentration as dependent variables. Furthermore, post hoc Tukey’s HSD test in SPSS was used to analyze the effect of the treatment on the root morphology and nutrient accumulation for each compartment separately.

In addition, the effects of nutrient accumulation in shoots were measured by using ANOVA with competition (with competitor *vs*. without competitor), nutrient heterogeneity (homogeneous *vs*. heterogeneous), and fertilization (no fertilizer *vs*. fertilizer) as fixed factors and the nutrient accumulation in shoots as the dependent variable. Post hoc Tukey’s HSD test in SPSS was used to analyze the effect of treatment on nutrient accumulation in shoots.

We also used the ratio between the value of the different order root morphology variables in the competitive compartment and those in the non-competitive compartment of the same pot (e.g., SRL _ratio_ = SRL_competitive half_/SRL _non-competitive half_ for SRL ratio) to evaluate the root response to competition for each treatment in sub-experimentII[21]. A *t*-test was performed to analyze the difference between the ratio and a value of 1 based on a comparison of the confidence intervals of the ratio. When the ratio was equal to 1, we considered that root growth was symmetrical and unaffected by its neighbors. A ratio <1 indicated that competition had a negative effect on roots, and a ratio >1 indicated that competition had a positive effect on roots.

Finally, to measure the correlation of nutrient concentration with root morphology when a competitor and nutrient heterogeneity were present simultaneously, a bivariate correlation was conducted to analyze the correlation between the accumulation of root nutrients and root morphology in non-competitive compartments and competitive compartments of sub-experimentII, respectively. All statistical tests were performed using SPSS version 11.5.

## Results

### Root morphology

Statistical analysis demonstrated that the SRL, SRA and density of first-order roots between compartments were significantly different (Table 1). Fertilization decreased the SRL, SRA, diameter and increased density of the second-order roots, as well as decreased the SRL, SRA of third-order roots. Although nutrient heterogeneity did not affect the SRL of first-order roots, its interaction with competition negatively affected the SRL of first-order roots (*F* = 4.40, *p*<0.05). Competition significantly enhanced the SRL, SRA, and diameter of first-order roots (*F* = 17.41, *p*<0.001; *F* = 14.73, *p*<0.001; *F* = 6.99, *p*<0.01, respectively).

**Table 1:** Factorial ANOVA results (F values) of fine root morphology at different branch orders in the sub-experiment I and II affected by fertilization, competition, nutrient heterogeneity and compartment, as well as their interactions.

*Between-subjects effects*	*df*	*F-value (First order)*				*F-value (Second order)*				*F-value (Third order)*			
		SRL	SRA	Diameter	Density	SRL	SRA	Diameter	Density	SRL	SRA	Diameter	Density
**Compartment**		5.20*	11.23**	0.05	9.15**	0.00	0.02	0.11	0.20	1.71	0.36	1.01	1.28
**Fertilization**	1	4.22*	2.96	2.21	0.02	16.94***	15.77***	7.62**	6.50*	5.25*	4.65*	3.75	2.34
**Heterogeneity**	1	1.96	4.22*	0.19	2.35	0.69	0.73	0.41	0.03	2.74	1.29	1.90	0.05
**Competition**	1	17.41***	14.73***	6.99**	2.28	2.53	3.63	0.14	2.40	0.00	0.01	0.44	0.09
Compa*F	1	4.41*	2.74	3.79	0.00	0.85	0.47	1.06	0.08	0.96	1.65	0.02	5.87*
Compa*H	1	1.76	3.21	0.30	1.07	0.27	0.37	0.05	0.64	2.49	1.64	0.47	0.10
F* **H**	1	0.71	0.36	0.87	0.01	0.56	0.41	0.00	0.02	0.07	0.00	0.68	0.16
Compa*F*H	1	0.95	1.82	0.08	1.68	0.04	0.01	0.03	0.08	2.19	0.72	0.70	0.63
Compa*Compe	0												
F*Compe	1	1.30	0.66	0.93	0.30	0.67	1.19	0.14	0.41	0.84	1.95	0.27	2.37
Compa*F*Compe	0												
H*Compe	1	4.40*	3.83	1.45	1.21	0.63	1.25	0.05	0.39	0.01	0.03	0.43	0.79
Compa*H*Compe	0												
F*H*Compe	1	0.57	0.15	3.35	2.02	0.03	1.21	2.01	4.42*	1.59	1.11	0.27	0.58
Compa*F*H*Compe	0												

Significance:

***p<0.001.

**P<0.01.

*p<0.5.

Compa: Compartment; Compe: Competition; F: fertilization; H: heterogeneity.

**Fig 2:**
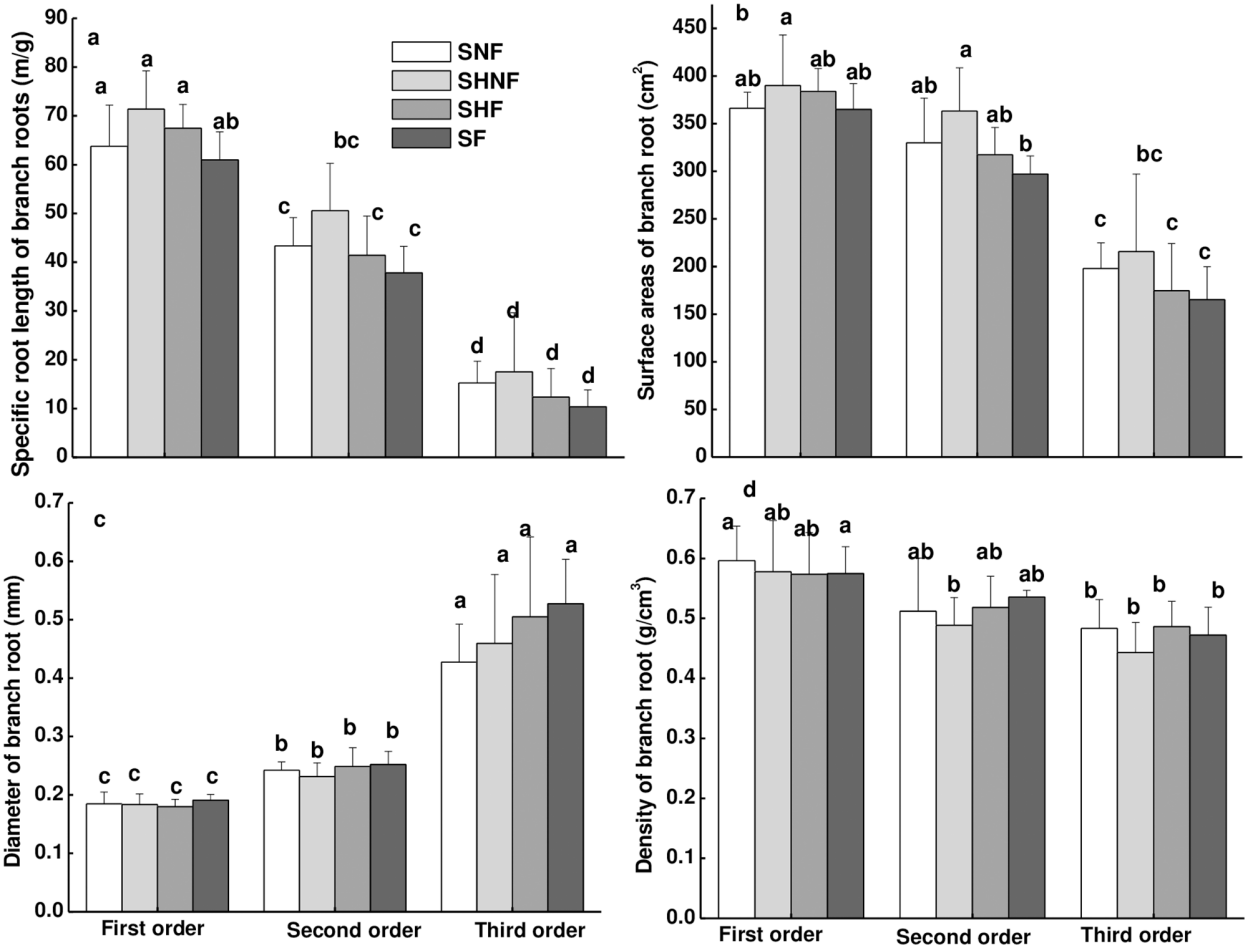
Fine root morphology (SRL (a), SRA (b), Diameter (c), Tissue density (d)) at different branch order affected by nutrient heterogeneity. Different letters indicate significant treatment effect between the means at *p*<0.05 analyzed by post hoc Tukey’s HSD test (means ± SE, n = 8).

Both in the control (SNF treatment) and SF treatment, the fine root morphologic parameters between both compartments of the same container were not significantly different in a homogeneous soil environment (S1 Table). Thus, we only present root morphologic data of half of the container in Fig 2. When the targeted plant was placed in the container in the absence of competitor (sub-experiment I), SRL, SRA and tissue density decreased with increasing branch order, but the root diameter increased with increasing branch order. The treatment (the SNF, SHF and SF treatments) had no effect on the SRL, SRA, root diameter or tissue density of any of the branch order roots (Fig 2).

**Fig 3:**
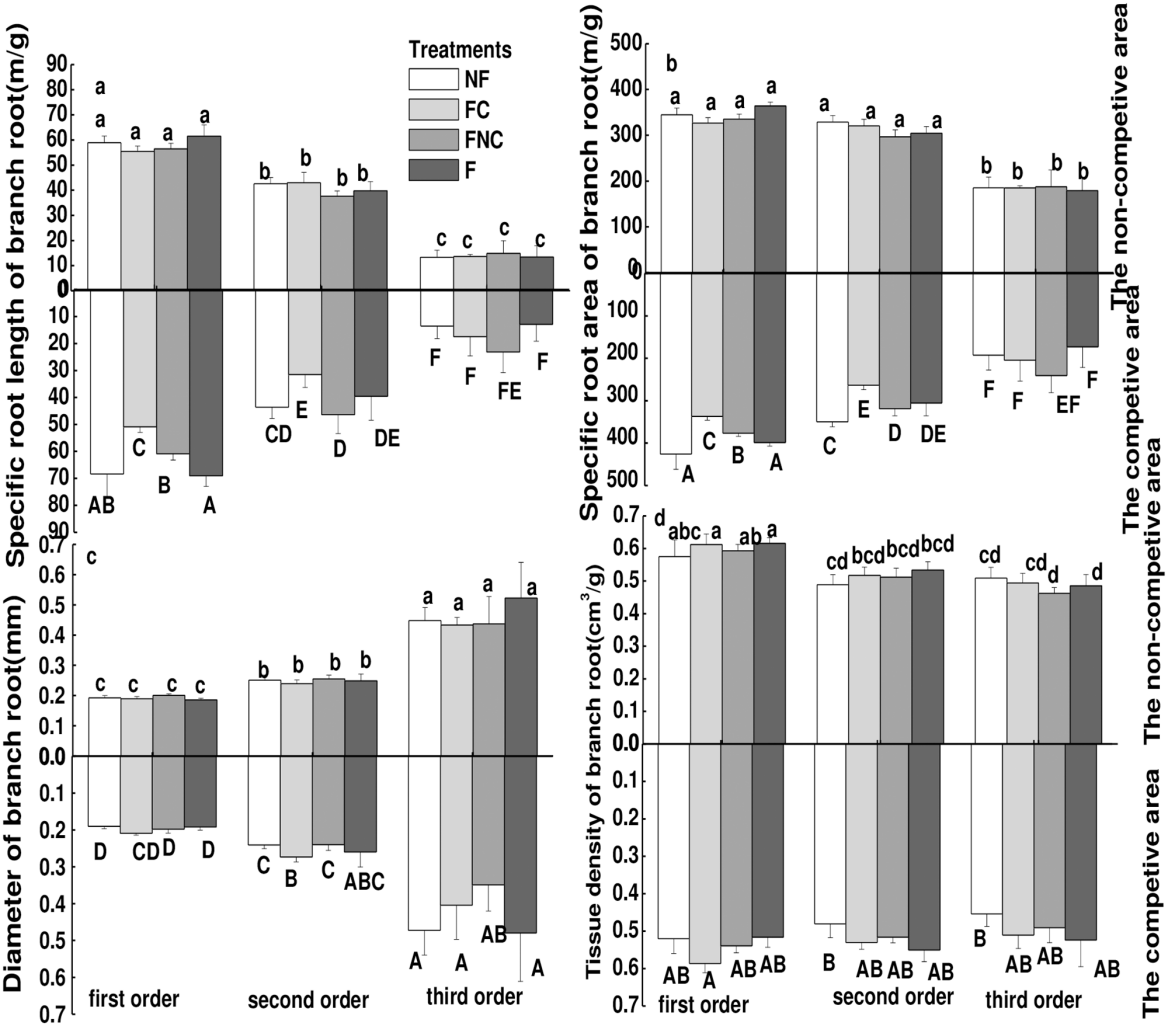
Fine root morphology (SRL (a), SRA (b), Diameter (c), Tissue density (d)) at different branch order affected by the competition and its interaction with nutrients heterogeneity in the competitive and non-competitive compartments. Different lowercase letters (eg. a, b, c) indicate significant treatment effect between the means in the non-competitive and the capital letters (eg. A, B, C) indicate significant treatment effect between the means in the competitive compartment at *p*<0.05 analyzed by post hoc Tukey’s HSD test (means ± SE, n = 8).

When the targeted plant was placed in a container in the presence of competitors (sub-experimentII), as shown from the comparisons between the NF and FC treatments and between the FNC and F treatments in Fig 3 and S2 Table, in the non-competitive compartment, nutrient heterogeneity had no effect on the SRL, SRA, and tissue density of any of the branch order roots both in the nutrient-rich patch and nutrient-normal patch. Heterogeneity decreased the SRA of first- and second-order roots and posed no effects on the SRL, diameter and tissue density of any of branch orders roots in the nutrient-normal patch, as seen from the comparison between the FNC and NF treatments in the competitive compartment (Fig 3). However, in the nutrient-rich patch, nutrient heterogeneity decreased the SRL and SRA of first-order roots and increased the tissue density and diameter of first-order roots, as seen from the comparison between the FC and F treatments in the competitive compartment (Fig 3).

Furthermore, the SRL and SRA were more enhanced in the presence of competitors than in the absence of competitors in a homogeneous environment, as seen from the comparison between the NF treatment in the competitive compartment in Fig 3 and SNF treatment in Fig 2 as well as between the F treatment in the competitive compartment in Fig 3 and SF treatment in Fig 2. The calculated ratios of the SRL and SRA (SRL_ratio_ and SRA_ratio_) for roots of different orders between the competitive and non-competitive compartments of the same containers showed that SRL_ratio_ and SRA_ratio_ were significantly >1 for first- and second-order roots in all treatments, except for the FC treatment (Table 2). The diameter_ratio_ of first- and second-order roots was >1 in the FC treatment; the diameter_ratio_ was not significantly different from 1 in all other treatments. By contrast, the tissue density_ratio_ of first-order roots was <1 in the NF, FC and F treatments. Additionally, in a heterogeneous environment, competition decreased SRL and SRA according to the comparison between the FC treatment in Fig 3 and SHF treatment in Fig 2. In a heterogeneous environment, both the SRL and SRA ratios of first- and second-order roots were <1 and >1 in the FC and FNC treatments, respectively (Table 2).

**Table 2:** The ratio between the value of the root morphology variables at different order in the competitive compartment and the value in the non-competitive compartment in the sub-experiment II (e.g. SRL _ratio_ = SRL_competitive half_ /SRL_non-competitive-half_ for specific root length ratio).

	First order				Second order				Third order			
Treatments	SRL	SRA	Diameter	Density	SRL	SRA	Diameter	Density	SRL	SRA	Diameter	Density
**NF**	1.25±0.21*	1.22±0.13*	0.98±0.07	0.85±0.06*	1.22±0.13*	1.12±0.08*	0.92±0.08	0.98±0.12	0.82±0.47	1.31±0.51	0.90±0.23	0.94±0.13
**FC**	0.84±0.08*	0.97±0.09	1.15±0.01*	0.91±0.08*	0.74±0.21*	0.88±0.16	1.21±0.09*	0.98±0.08	1.21±0.57	1.08±0.29	0.96±0.20	1.02±0.11
**FNC**	1.20±0.16*	1.12±0.04*	0.95±0.10	0.95±0.10	1.23±0.17*	1.10±0.07*	0.90±0.10	1.04±0.16	2.07±1.62	1.35±0.66	0.86±0.34	1.02±0.19
**F**	1.22±0.11*	1.18±0.08*	0.98±0.04	0.87±0.06*	1.41±0.30*	1.19±0.15*	0.85±0.11*	1.03±0.16	1.55±0.79	1.20±0.31	0.84±0.22	1.07±0.23

Significance between the ratio and the value 1.00 analyzed by *t*-test:

* p <0.05.

SRL: specific root length, SRA: specific root area.

### Nutrients in roots

**Fig 4:**
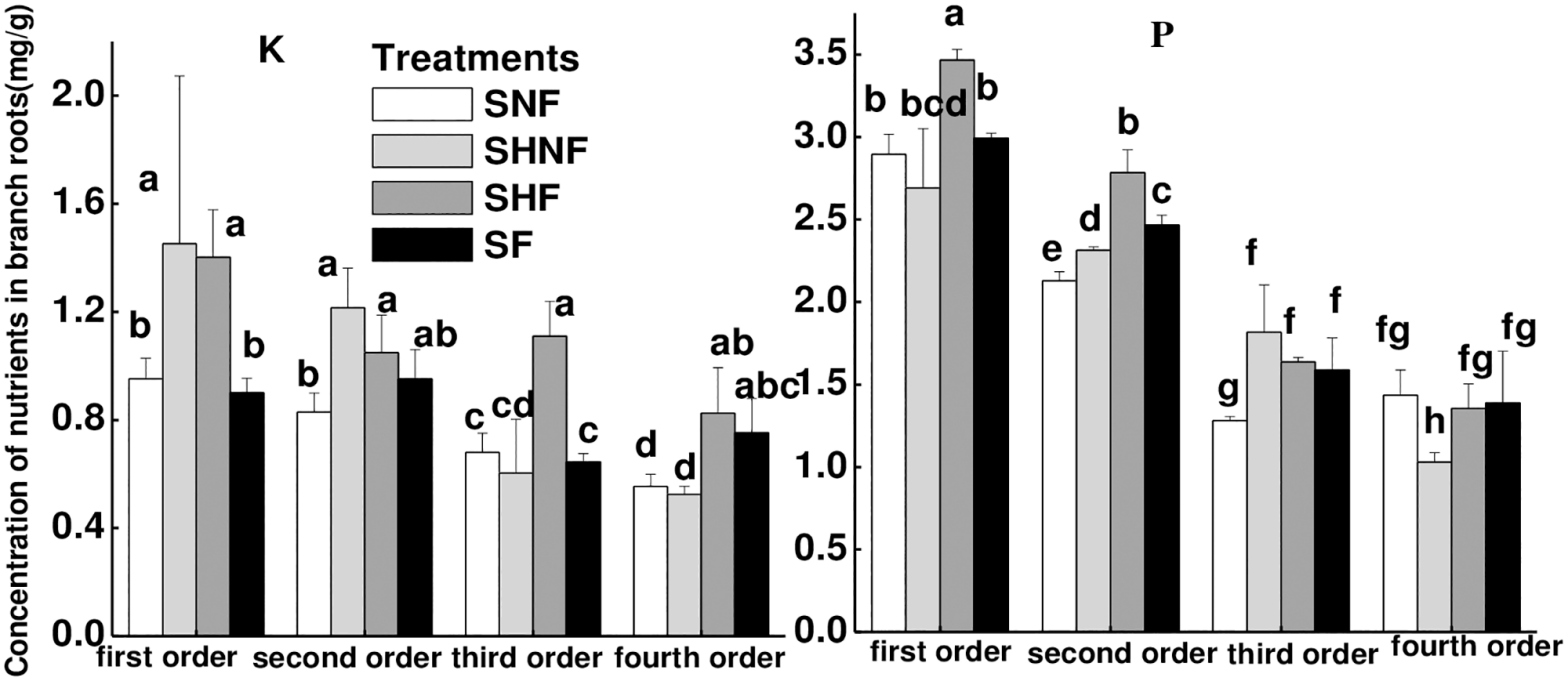
The concentrations of K and P in roots of different branch order affected by nutrients heterogeneity. **Noted**: SHNF is the non-fertilizer compartment in SHF treatment; SHF is the fertilizer compartment in SHF treatment. Different letters indicate significant treatment effects between the means at *p*<0.05 analyzed by post hoc Tukey’s HSD test (means ± SE, n = 8).

The statistical results show the factors, including the compartment, fertilization, nutrient heterogeneity and competition affected the concentration of K and P in roots (Table 3). When the targeted plant was placed in a container without competitors (sub-experiment I), as in the control, the concentrations of K and P decreased with increasing branch order in the SNF treatment (Fig 4 and S3 Table). The effects of fertilization or soil nutrient heterogeneity on the accumulation of nutrients in roots of different branch orders varied. Specifically, as demonstrated by the comparison between the SNF and SF treatments in Fig 4, fertilization increased the concentration of K in fourth-order roots and concentration of P in second- and third-order roots in a homogeneous environment. When the targeted plant was subjected to nutrient heterogeneity, there was an increase in the K concentration in first- and second-order roots and in the P concentration in second- and third-order roots in the SHNF compartment compared with the SNF compartment. Additionally, there was an increase of the K concentration in first- and third-order roots and in the P concentration in first- and second-order roots, as seen in the SHF compartment compared with the SF compartment (Fig 4).

**Table 3:** Factorial ANOVA results (*F* values) of nutrient concentration in roots of different branch order in the sub-experiment I and II affected by fertilization, competition, nutrient heterogeneity and compartment, as well as their interactions.

*Between-subjects effects*	*df*	K concentration in root *F-value*				P concentration in root *F-value*			
		first	second	third	fourth	first	second	third	fourth
**Compartment**		5.42*	3.25	3.67	24.35***	0.43	7.90**	0.22	26.1***
**Fertilization**	1	1.79	5.89*	13.16**	1.08	52.8***	13.63**	13.84**	1.35
**Heterogeneity**	1	26.0***	2.95	8.73**	0.00	11.08**	2.01	4.49*	0.05
**Competition**	1	6.15*	2.65	10.72**	9.90**	6.41*	3.98	0.30	7.46*
Compa*F	1	3.80	2.13	34.92***	0.02	0.58	0.41	0.39	0.64
Compa*H	1	2.22	1.77	0.24	0.03	0.01	2.17	0.36	0.05
F* **H**	1	1.91	0.06	0.10	0.15	2.84	5.79*	3.76	0.68
Compa*F*H	1	6.66*	11.51**	2.04	0.74	0.51	0.70	0.75	1.61
Compa*Compe	0	.	.	.	.	.	.	.	.
F*Compe	1	10.19**	6.45*	12.08**	0.81	0.03	6.02*	4.39*	0.08
Compa*F*Compe	0	.	.	.	.	.	.	.	.
H*Compe	1	1.49	5.20*	2.55	0.33	1.39	0.57	0.70	0.27
Compa*H*Compe	0	.	.	.	.	.	.	.	.
F*H*Compe	1	1.88	2.40	0.13	1.48	1.47	0.00	3.27	0.36
Compa*F*H*Compe	0								

Significance:

***p<0.001.

**P<0.01.

*p<0.5.

Compa: Compartment; Compe: Competition; F: fertilization; H: heterogeneity.

**Fig 5:**
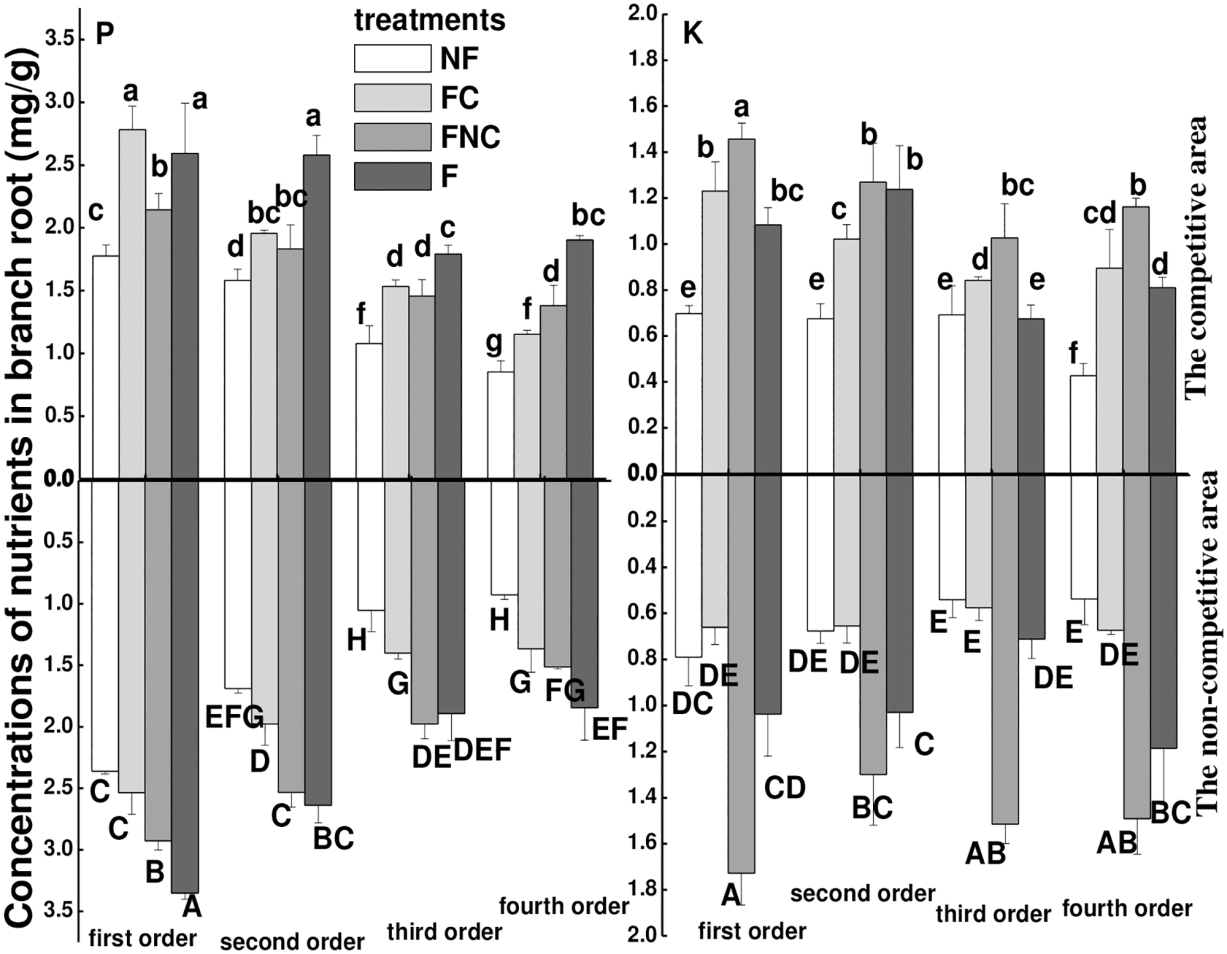
The concentrations of K and P in roots of different branch order affected by the competition and its interaction with nutrients heterogeneity in the competitive and non-competitive compartments. Different lowercase letters (eg. a, b, c) indicate significant treatment effects between the means in the non-competitive and the capital letters (eg. A, B, C) indicate significant treatment effects between the means in the competitive compartment at *p*<0.05 analyzed by post hoc Tukey’s HSD test (means ± SE, n = 8).

When the targeted plant was placed in a container with competitors (experimentII), as shown by the comparison between the F and NF treatments in Fig 5 and S4 Table, fertilization enhanced the concentration of K and P in roots of all branch orders in both the competitive and non-competitive compartments. In the competitive compartment, nutrient heterogeneity increased the concentration of K and P of roots of all branch orders in the nutrient-normal pot from the comparison between FNC and NF treatments compared with the nutrient concentration in equivalent patches under a homogenous environment (Fig 5). However, in a nutrient-rich pot in the competitive compartment, nutrient heterogeneity decreased the P concentration of roots of all branch orders except for the first-order roots, as seen from the comparison between the FC and F treatments. K in the nutrient-rich pot in the competitive compartment was only decreased in second-order roots in a nutrient heterogeneous environment compared with a homogeneous environment (Fig 5). Furthermore, competition significantly affected the concentrations of P and K in first- and fourth-order roots (Table 3). When comparing the nutrient concentrations in the roots of all branch orders between the competitive and non-competitive compartments of the same container, the concentrations of P were higher in the non-competitive compartment than in the competitive compartment, except in the FC treatment (Fig 5). This was not the case with the root K concentration.

Finally, the P concentration in all branch order roots was positively correlated with SRL, SRA and root density, but negatively correlated with root diameter, both in the competitive and non-competitive compartments. The K concentration was not associated with root morphology (Table 4).

**Table 4:** Bivariate correlation coefficients (r^2^) of fine root morphology and nutrient concentration in the competitive and non-competitive compartments respectively.

		SRL	SRA	Diameter	Density	K
**Non-competitive area**	**SRA**	0.974***				
	**Diameter**	-0.962***	-0.978***			
	**Density**	0.847***	0.732**	-0.756**		
	**K**	0.209	0.175	-0.213	0.129	
	**P**	0.781**	0.729**	-0.717**	0.741**	0.572*
**Competitive area**	**SRA**	0.985***				
	**Diameter**	-0.931***	-0.943***			
	**Density**	0.406	0.335	-0.494		
	**K**	0.428	0.360	-0.552	0.614*	
	**P**	0.603*	0.532	-0.645*	0.861***	0.643*

Significance:

*** p<0.001.

** P<0.01.

* p<0.5.

SRL: specific root length, SRA: specific root area.

### Nutrients in shoots

**Fig 6:**
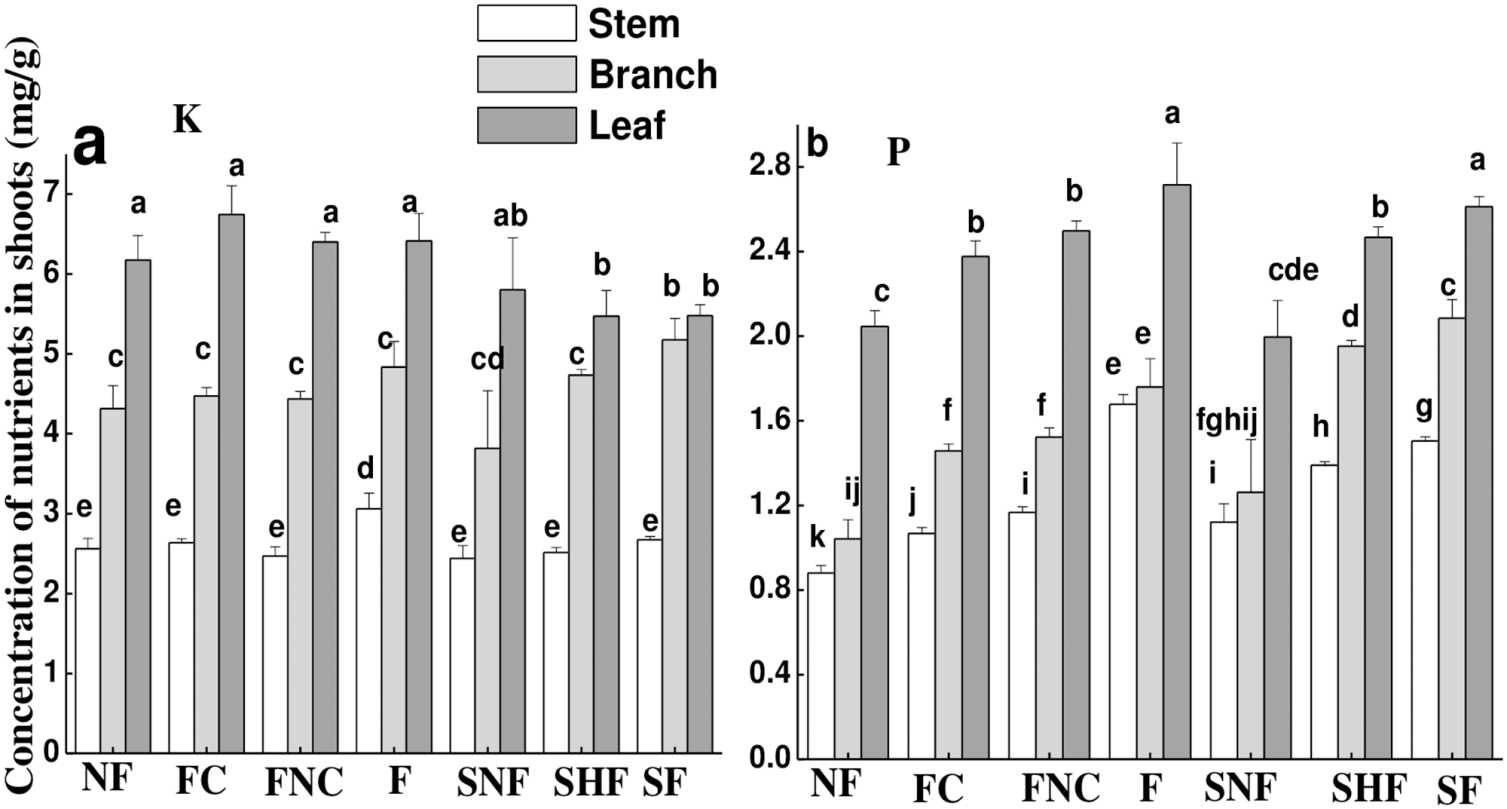
The concentrations of K (a) and P (b) in shoots of different parts under different treatments. Different letters indicate significant treatment effects between the means at *p*<0.05 analyzed by post hoc Tukey’s HSD test (means ± SE, n = 8).

The statistics showed that fertilization, nutrient heterogeneity and competition affected the concentrations of K and P in shoots to some extent (Table 5). Both K and P mainly accumulated in leaves (Fig 6 and S5 Table). The concentration of K in the branches of the targeted plant without competitors was increased after fertilization, whereas the K concentration in leaves of plants both in the presence and absence of competitors was not affected by fertilization (Fig 6a). The concentration of K in leaves of plants was higher in the presence of competitors than in the absence of competitors. However, competition did not affect the P concentration in leaves. The concentration of P in all stems, branches and leaves was increased by fertilization (Fig 6b). With regard to soil heterogeneity, only the P concentration in leaves and K concentration in stems were influenced.

**Table 5:** Factorial ANOVA results (*F* values) of nutrient concentration at different parts of shoots in the sub-experiment I and II affected by fertilization, competition or nutrient heterogeneity, as well as their interactions.

*Between-subjects effects*	*df*	K in *shoots (F-value)*			P in *shoots (F-value)*		
		stems	branches	leaves	stems	branches	leaves
**Fertilization**	1	24.25***	24.72***	0.04	381***	130***	94.8***
**Heterogeneity**	1	23.19***	5.44*	0.15	142***	10.09**	11.77**
**Competition**	1	5.69*	0.02	21.47***	49.76***	39.08***	0.30
F* **H**	0						
F*C	1	3.23	4.92*	1.82	47.14***	0.60	0.17
H*C	1	6.33*	0.03	0.18	62.51***	1.20	1.17
F*H*C	0						

Significance:

***p<0.001.

**P<0.01.

*p<0.5.

C: Competition; F: fertilization; H: heterogeneity

## Discussion

### Responses of fine root morphology

In the control treatment (SNF treatment) of sub-experiment I, there was no difference in the fine root morphology between the two compartments of the container (S1 Table). Thus, we ignored the influence of the experimental pot and systematic error when evaluating the effects of competition or soil nutrient heterogeneity on the root morphology. In addition, the responses of roots were only due to the experimental treatments.

Fine roots displayed different functions at different branch orders and were most sensitive to environmental changes among the whole root system [23,34]. In addition, lower order roots mainly absorbed nutrients and were more sensitive to the environment than higher order roots. Fine root morphology incorporates fundamental root system traits that control the assimilation of soil water and nutrients. To date, although several studies in the literature have revealed the profound consequences of soil heterogeneity, competition and nutrient heterogeneity–competition interactions for plants in agroecosystems [35], forests [24,25] and grasslands [11,21], these studies mainly investigated the responses of root production or biomass to soil heterogeneity and/or competition [6]. The measures of root biomass are not necessarily indicative of the total absorptive area of the root system, and any changes in the root morphology can occur without a change in the total root biomass. Fransen et al. [17,36] and Mommer et al. [37] reported that *F*. *rubra* showed increased biomass in nutrient-rich patches than *A*. *odoratum*, but had lower root length densities with lower specific root lengths than *A*. *odoratum* as well as acquired fewer nutrients. Moreover, the specific root lengths of both aforementioned plants were higher in a nutrient-rich patch than a nutrient-poor patch. However, the present study indicated that the SRL and SRA of all branch order roots were not significantly different between nutrient-rich patches and nutrient-normal patches in a heterogeneous environment (Fig 2a and 2b). Meanwhile, when the plant was growing in a homogenous environment without competitors, the addition of fertilization did not affect the root morphology, including the SRL, SRA, root diameter and tissue density (between the SNF and SF treatments in Fig 2) of the targeted fine roots. From the above two results, we inferred that when there were sufficient soil nutrients to maintain plant growth, the root morphology remained unchanged. In a previous work by our team, Nan et al. [31], we reported that the spruce root architecture (the number of root tips over root surface, RTRS and root order length percentage, ROLP) increased in nutrient-rich patches, which showed a different root morphology response to the present result. This indicated that the root architecture and root morphology respond to soil nutrients differently.

Furthermore, significantly greater root proliferation has been observed in previous studies when a fixed supply of nutrients is provided heterogeneously to single plants than in equivalent patches in homogenous treatments [9–10,17,36]. Nan et al. [31] observed that a target plant that received the FV treatment (only applying fertilizer in the competitive half) had a higher ROLP (root architecture) and biomass in first-order roots in the non-competitive half compared with the NF treatment (applying no fertilizer both in the two halves) as well as a higher ROLP in third-order roots in the non-competitive half compared with the F treatment (applying fertilizer both in the two halves). They reported that plants simultaneously exposed to nutrient heterogeneity and neighboring plants attempted to increase their root architecture regardless of the distribution of resources. By contrast, in the present study, nutrient heterogeneity had no effect on the SRL and SRA of all branch order roots either in nutrient-normal or nutrient-rich patches in the absence of competitors (Fig 2a and 2b), but reduced the SRL and SRA of first-order roots in nutrient-rich patches in the presence of competitors (between the FC treatment and F treatment in competitive compartments, as shown in Fig 3a and 3b, respectively). There was also no effect on the root diameter and tissue density in the absence of competitors (Fig 2c and 2d), but the nutrient heterogeneity increased the diameter and tissue density of first-order roots in the nutrient-rich patch in the presence of competitors (Fig 3c and 3d).

Combined, these two results mean that nutrient heterogeneity led spruce seedlings to produce an increased root biomass with a smaller root system size (including lower root length, surface area and volume) based on the formula tissue density = biomass/root volume = biomass/ (root length*surface areas) in the competitive compartment, which also implied that the presence of competitors limited the soil space for root spreading, but did not affect root growth in a heterogeneous environment. Janecek et al. [13] investigated the effects of nutrient heterogeneity and competition on the biomass of *C*. *hartmanii* and *M*. *caerules* and observed non-additive effects: a positive response to nutrient heterogeneity disappeared when competition from other species existed. Cahill et al. [11] also found non-additive effects of intraspecific competition and nutrient heterogeneity on the horizontal spread of roots. Briefly, although nutrient heterogeneity restrained the development of the root morphology, it did not negatively affect spruce growth either in the presence or absence of competitors in this study.

As discussed above, soil nutrients did not affect the root morphology, so how did competition from neighboring roots affect the root morphology? Nan et al. [31] investigated the response of the spruce root architecture to competition and showed that under combinations of homogenous nutrients and root competition, target plants adopted the strategy of decreasing the length percentage ratio of diameter-based fine root subclasses to total fine root (SRLP) in 0–0.5 mm fine roots to alleviate competition. In this study, competition enhanced the SRL and SRA of first- and second-order roots both in fertilization and non-fertilization treatments in a homogenous soil environment, which indicated an adaptation strategy to avoid competition for resources by neighbors by increasing SRL and SRA to take up more nutrients [24]. This is also a foraging strategy of spruce, which tends to increase the efficiency of soil exploitation and space sequestration in soil layers that are less occupied by competitors [25]. A similar result was reported in the presence of interspecific competition [24]. Furthermore, root tissue density was greater in the non-competitive compartment than in the competitive compartment, regardless of fertilization and soil heterogeneity. This result also indicated that competition decreased the biomass and enlarged the root volume by increasing the root length and area, which were consistent with the above results of the responses of SRL and SRA to competition.

Moreover, Coomes et al. [38] reported that resource competition is most significant when resources are abundant and that the intensity of competition strengthens as soil resources increase in a northern hardwood forest [39]. However, in our study, competition was not affected by the application of fertilizer. In addition, in a heterogeneous environment, Mommer et al. [12] reported that competition resulted in an increased root length in empty patches rather than selective proliferation because of neighboring plants competing for resources in a nutrient-rich patch. In this study, in a heterogeneous environment, competition decreased SRL and SRA (between FC and SHF treatments) and showed an antagonistic effect with nutrient heterogeneity, i.e., a positive response to competition became negative due to nutrient heterogeneity. Fransen et al. [17] investigated the responses of two perennial grass species to competition in the presence of nutrient heterogeneity and found that nutrient heterogeneity enhanced the competitive ability of *A*. *odoratum*. Finally, in a heterogeneous environment, the ratio values of SRL and SRA of the former two orders of roots were <1 and >1 in the FC and FNC treatments, respectively, which showed that lower SRL and SRA were observed in nutrient-rich patches than in nutrient-normal patches when competitors were present. This may be the reason that fertilization offset nutrients for which the competitors’ roots competed, leading to the disappearance of the foraging strategy induced by competition, which also implied that the root morphological features were only stimulated when the soil resources were insufficient for plant growth.

### Nutrient capture mediated by the fine root morphology?

Past studies suggested that the root morphology, such as SRL and SRA, are important to water and nutrient uptake for plants and can be used as useful indicators of the nutrient capture of roots. In present study, based on the above discussion, the root morphology just was an indicator for some nutrient. For instance, the fine root morphology, such as SRL, SRA, root diameter and tissue density, was only linearly correlated with the P concentration, not with the K concentration in varied branch roots, which suggested that root morphology can indicate P capture but was not an indicator for K nutrient capture. How shifts in root morphology drive changes in nutrient capture when the plant is subjected to competition or nutrient heterogeneity? In this study, the addition of fertilization did not affect SRL and SRA, but the P and K concentrations of all branch orders roots were still enhanced under homogenous soil in absence or presence of competition. And in heterogeneous soil, the root P and K concentrations were greater in nutrient-rich patches than in nutrient-normal patches, but the root morphology showed no difference, which proved that the soil nutrients were sufficient for the plant requirements and the root morphology was not affected by fertilization. This also suggested that the root morphology did not mediate nutrients capture under enough soil nutrients irrespective of heterogeneous or homogeneous soil.

However, roots capture nutrients efficiently by increasing SRL and SRA of first- and second-order roots both in fertilization and non-fertilization treatments when plants are subjected to competition for soil resources from neighboring plants. For example, although the effect of competition on the K concentration in roots was not significant, the K concentration in leaves was enhanced by competition both in homogenous and heterogeneous environments, which suggested that the increased capture of K by roots was transported to shoots in the presence of competition. On the other hand, increasing SRL and SRA would enhance the uptake efficiency of the roots, but could not offset the influence of the lack of nutrients in soil induced by competition and ultimately competition led to a reduced P concentration in roots. All this result showed that the root morphological features were stimulated to mediate nutrients capture by root when the soil nutrients were insufficient for plant growth and the nutrients uptake mainly affected by the soil nutrients when plants were subjected to nutrient heterogeneity and competition.

In addition, when the nutrient heterogeneity interacts with competition on plant, competition decreased SRL and SRA but enhanced the capture of K. and in the nutrient-normal patch, nutrient heterogeneity only slight decreased the SRA of first- and second-order roots and posed no effects on the SRL, diameter and tissue density of any of branch orders roots in the competitive compartment, but nutrient heterogeneity increased the concentration of K and P of roots of all branch orders compared with the nutrient concentration in equivalent patches under a homogenous environment. These two results also suggested that the nutrients capture by root were mainly affected by the soil nutrients more than the root morphology. Over all, these results showed that root morphology is a limited indicator for nutrient capture [40] and proved that root morphology was a mediator for nutrient capture and plant growth in some special environment, i.e., a decrease in soil resources or less bioavailability of nutrients.

On the other hand, in the present study and previous study conducted by Nan et al. [31], it was shown that the response of nutrient concentration in root to competition and resource distribution in soil in the present study were similar to the response of the root architecture [31], although we could not analyze the correlation between nutrient concentration in roots and the root architecture because these data were not from same experiment. This shows that in previous studies, the root architecture was an indicator of the root foraging ability. Previous studies lead to the misunderstanding that the root morphology and root architecture are similar physiological functions concerning nutrient uptake. The combination of the present study and previous study conducted by Nan et al. [31] showed the different functions between the root morphology and root architecture when plants were subjected to changes in the soil environment. The results of the present study provide a further understanding of the influences of competition and soil nutrient heterogeneity on the growth of woody plants.

## Conclusion

In conclusion, root morphology is sensitive to changes in resources in the soil environment and can mediate nutrient uptake by developing a root system when plants are subjected to competition. When the soil nutrients were sufficient for plant growth, the root morphology was not significantly affected by fertilization and soil heterogeneity when competition was absent. However, the P and K concentrations in all branch order roots were higher in nutrient-rich soils than in nutrient-normal soils, and nutrient heterogeneity increased the P and K concentrations of the first three orders of roots compared with those of roots in equivalent patches under a homogenous environment in the absence of competition. Furthermore, *P*. *asperata* avoided competition for resources and space with competitors and facilitated the efficient uptake of nutrients by increasing the SRL and SRA of first- and second-order roots and decreasing the tissue density of first-order roots when the soil resources were insufficient for plant growth. On the interaction between nutrient heterogeneity and competition, competition decreased the SRL and SRA but enhanced the capture of K under heterogeneous soil compared with under homogeneous soil. The nutrients captures by root were mainly affected by the soil nutrients more than the root morphology. Combined with a previous study of our team [31], plants showed different responses to nutrient heterogeneity and competition between the root morphology and root architecture. We believe that our research findings will strengthen the understanding of the mechanisms of heterogeneity and competition that affect plant growth.

## Supporting information

S1 Table Fine root morphology (SRL, SRA, Diameter, Tissue density) at different branch order affected by nutrient heterogeneity in the two compartments (means ± SE, n = 8). Note: for SNF and SF, the two compartments were under same soil condition.
(DOCX)

S2 Table Fine root morphology (SRL, SRA, Diameter, Tissue density) at different branch order affected by the competition and its interaction with nutrients heterogeneity in the competitive and non-competitive compartments (means ± SE, n = 8). (DOCX)

S3 Table The concentrations of K and P in roots of different branch order affected by nutrients heterogeneity (means ± SE, n = 8). Note: for SNF and SF, the two compartments were under same soil condition.
(DOCX)

S4 Table The concentrations of K and P in roots of different branch order affected by the competition and its interaction with nutrients heterogeneity in the competitive and non-competitive compartments (means ± SE, n = 8). (DOCX)

S5 Table The concentrations of K and P in roots of different branch order affected by the competition and its interaction with nutrients heterogeneity in the competitive and non-competitive compartments (means ± SE, n = 8). (DOCX)
