# Supplementary material for: Responses of nutrient capture and fine root morphology of subalpine coniferous tree Picea asperata to nutrient heterogeneity and competition
Source: PLoS One. 2017 Nov 2;12(11):e0187496. doi: 10.1371/journal.pone.0187496 (PMC5667764; doi:10.1371/journal.pone.0187496)
Supplement: S1 Table — Note: for SNF and SF, the two compartments were under same soil condition. (DOCX) [file pone.0187496.s001.docx]

**S1 Table. Fine root morphology (SRL, SRA, Diameter, Tissue density) at different branch order affected by nutrient heterogeneity in the two compartments (means + SE, n=8). Note: for SNF and SF, the two compartments were under same soil condition.**

|  |  | **Compartment / fertilizer in SHF** | | | | **Compartment/ no fertilizer in SHF** | | | |
| --- | --- | --- | --- | --- | --- | --- | --- | --- | --- |
|  | **Treatments** | **SRL**  **(cm/g)** | **SRA**  **(cm^2^/g)** | **Diameter**  **(mm)** | **Density**  **(g/cm^3^)** | **SRL**  **(cm/g)** | **SRA**  **(cm^2^/g)** | **Diameter**  **(mm)** | **Density**  **(g/cm^3^)** |
| **First-**  **order root** | **SNF** | 6380+840 | 366+16.5 | 0.185+0.020 | 0.596+0.58 | 6562+935 | 370+22.0 | 0.181+0.020 | 0.607+0.058 |
|  | **SHF** | 6750+482 | 384+24.0 | 0.180+0.012 | 0.573+0.07 | 7139+787 | 390+53.3 | 0.183+0.019 | 0.578+0.086 |
|  | **SF** | 6095+576 | 365+27.1 | 0.191+0.010 | 0.575+0.045 | 5962+491 | 356+28.0 | 0.191+0.005 | 0.587+0.044 |
| **Second-order root** | **SNF** | 4334+582 | 330+46.8 | 0.242+0.014 | 0.512+0.088 | 5189+508 | 366+16.7 | 0.225+0.010 | 0.489+0.042 |
|  | **SHF** | 4145+803 | 317+28.4 | 0.249+0.032 | 0.518+0.052 | 5059+968 | 363+45.3 | 0.231+0.023 | 0.489+0.047 |
|  | **SF** | 3784+542 | 297+19.1 | 0.252+0.022 | 0.536+0.011 | 3582+622 | 290+24.3 | 0.261+0.024 | 0.532+0.010 |
| **Third-**  **order root** | **SNF** | 1525+446 | 198+27.0 | 0.428+0.065 | 0.483+0.048 | 1772+710 | 228+65.4 | 0.430+0.094 | 0.478+0.121 |
|  | **SHF** | 1234+587 | 175+49.4 | 0.505+0.137 | 0.486+0.042 | 1651+908 | 216+81.3 | 0.460+0.118 | 0.443+0.050 |
|  | **SF** | 1036+346 | 165+34.5 | 0.527+0.076 | 0.472+0.046 | 1556+777 | 199+64.9 | 0.519+0.292 | 0.459+0.077 |
