# Supplementary material for: Responses of nutrient capture and fine root morphology of subalpine coniferous tree Picea asperata to nutrient heterogeneity and competition
Source: PLoS One. 2017 Nov 2;12(11):e0187496. doi: 10.1371/journal.pone.0187496 (PMC5667764; doi:10.1371/journal.pone.0187496)
Supplement: S2 Table — (DOCX) [file pone.0187496.s002.docx]

**S2 Table.** **Fine root morphology (SRL, SRA, Diameter, Tissue density) at different branch order affected by the competition and its interaction with nutrients heterogeneity in the competitive and non-competitive compartments (means + SE, n=8).**

|  |  | **Non-competitive compartment** | | | | **Competitive compartment** | | | |
| --- | --- | --- | --- | --- | --- | --- | --- | --- | --- |
|  | **Treatments** | **SRL**  **(cm/g)** | **SRA**  **(cm^2^/g)** | **Diameter**  **(mm)** | **Density**  **(g/cm^3^)** | **SRL**  **(cm/g)** | **SRA**  **(cm^2^/g)** | **Diameter**  **(mm)** | **Density**  **(g/cm^3^)** |
| **First-**  **order root** | **NF** | 5893+260 | 345+14.5 | 0.192+0.008 | 0.575+0.051 | 6839+873 | 426+35.8 | 0.190+0.006 | 0.520+0.039 |
|  | **FC** | 5543+214 | 327+11.9 | 0.189+0.007 | 0.612+0.032 | 5090+204 | 337+8.88 | 0.209+0.005 | 0.587+0.024 |
|  | **FNC** | 5645+223 | 335+11.2 | 0.201+0.005 | 0.593+0.020 | 6090+228 | 377+7.32 | 0.198+0.011 | 0.539+0.019 |
|  | **F** | 6153+443 | 364+8.4 | 0.185+0.005 | 0.616+0.017 | 6907+391 | 399+8.82 | 0.192+0.009 | 0.516+0.027 |
| **Second-order root** | **NF** | 4256+249 | 329+14.2 | 0.251+0.017 | 0.489+0.031 | 4358+419 | 350+11.8 | 0.241+0.01 | 0.481+0.037 |
|  | **FC** | 4297+418 | 321+14.3 | 0.239+0.012 | 0.517+0.025 | 3147+481 | 263+10.7 | 0.273+0.013 | 0.530+0.018 |
|  | **FNC** | 3758+211 | 297+15.2 | 0.255+0.013 | 0.512+0.027 | 4643+695 | 319+16.7 | 0.240+0.016 | 0.516+0.015 |
|  | **F** | 3976+367 | 304+14.5 | 0.248+0.023 | 0.534+0.026 | 3954+887 | 305+30.5 | 0.260+0.041 | 0.550+0.031 |
| **Third-**  **order root** | **NF** | 1333+277 | 185+23.5 | 0.448+0.043 | 0.509+0.033 | 1353+465 | 193+34.9 | 0.472+0.067 | 0.454+0.033 |
|  | **FC** | 1363+84.1 | 185+4.74 | 0.433+0.025 | 0.494+0.03 | 1745+713 | 204+49.4 | 0.404+0.093 | 0.511+0.036 |
|  | **FNC** | 1486+504 | 187+36.8 | 0.437+0.091 | 0.462+0.018 | 2318+759 | 241+40.4 | 0.349+0.071 | 0.491+0.04 |
|  | **F** | 1347+446 | 179+27.9 | 0.523+0.117 | 0.485+0.035 | 1287+623 | 173+48.9 | 0.480+0.131 | 0.524+0.071 |
