# Supplementary material for: Responses of nutrient capture and fine root morphology of subalpine coniferous tree Picea asperata to nutrient heterogeneity and competition
Source: PLoS One. 2017 Nov 2;12(11):e0187496. doi: 10.1371/journal.pone.0187496 (PMC5667764; doi:10.1371/journal.pone.0187496)
Supplement: S3 Table — Note: for SNF and SF, the two compartments were under same soil condition. (DOCX) [file pone.0187496.s003.docx]

**S3 Table. The concentrations of K and P in roots of different branch order affected by nutrients heterogeneity (means + SE, n=8). Note: for SNF and SF, the two compartments were under same soil condition.**

|  |  | **Compartment / fertilizer in SHF** | | | **Compartment / no fertilizer in SHF** | |
| --- | --- | --- | --- | --- | --- | --- |
|  | **Treatments** | **K**  **(mg/g)** | **P**  **(mg/g)** | **K**  **(mg/g)** | | **P**  **(mg/g)** |
| **First-**  **order root** | **SNF** | 0.952+0.075 | 2.896+0.119 | 0.970+0.017 | | 2.758+0.292 |
|  | **SHF** | 1.402+0.17 | 3.466+0.064 | 1.452+0.620 | | 2.690+0.361 |
|  | **SF** | 0.900+0.054 | 2.992+0.030 | 0.810+0.141 | | 2.278+1.035 |
| **Second-**  **order root** | **SNF** | 0.829+0.069 | 2.129+0.054 | 0.840+0.041 | | 1.912+0.272 |
|  | **SHF** | 1.049+0.139 | 2.784+0.139 | 1.215+0.147 | | 2.312+0.021 |
|  | **SF** | 0.952+0.108 | 2.467+0.059 | 1.056+0.015 | | 2.366+0.164 |
| **Third-**  **order root** | **SNF** | 0.680+0.071 | 1.282+0.023 | 0.789+0.265 | | 1.035+0.285 |
|  | **SHF** | 1.109+0.129 | 1.636+0.028 | 0.603+0.199 | | 1.817+0.286 |
|  | **SF** | 0.644+0.031 | 1.587+0.197 | 0.792+0.112 | | 1.548+0.579 |
| **Fourth-**  **order root** | **SNF** | 0.554+0.044 | 1.435+0.152 | 0.652+0.237 | | 1.378+1.023 |
|  | **SHF** | 0.824+0.168 | 1.355+0.149 | 0.525+0.029 | | 1.030+0.056 |
|  | **SF** | 0.752+0.126 | 1.387+0.314 | 0.643+0.181 | | 1.103+1.138 |
