# Supplementary material for: Responses of nutrient capture and fine root morphology of subalpine coniferous tree Picea asperata to nutrient heterogeneity and competition
Source: PLoS One. 2017 Nov 2;12(11):e0187496. doi: 10.1371/journal.pone.0187496 (PMC5667764; doi:10.1371/journal.pone.0187496)
Supplement: S4 Table — (DOCX) [file pone.0187496.s004.docx]

**S4 Table. The concentrations of K and P in roots of different branch order affected by the competition and its interaction with nutrients heterogeneity in the competitive and non-competitive compartments (means + SE, n=8).**

|  |  | **Non-competitive Compartment** | | | **Competitive Compartment** | |
| --- | --- | --- | --- | --- | --- | --- |
|  | **Treatments** | **K**  **(mg/g)** | **P**  **(mg/g)** | **K**  **(mg/g)** | | **P**  **(mg/g)** |
| **First-**  **order root** | **NF** | 0.790+0.123 | 2.360+0.022 | 0.696+0.035 | | 1.776+0.087 |
|  | **FC** | 0.660+0.075 | 2.535+0.175 | 1.231+0.127 | | 2.783+0.185 |
|  | **FNC** | 1.727+0.139 | 2.926+0.074 | 1.457+0.069 | | 2.144+0.128 |
|  | **F** | 1.037+0.182 | 3.351+0.049 | 1.083+0.075 | | 2.593+0.398 |
| **Second-order root** | **NF** | 0.677+0.053 | 1.691+0.034 | 0.675+0.065 | | 1.582+0.090 |
|  | **FC** | 0.654+0.073 | 1.976+0.173 | 1.021+0.063 | | 1.955+0.026 |
|  | **FNC** | 1.300+0.220 | 2.532+0.119 | 1.270+0.168 | | 1.833+0.189 |
|  | **F** | 1.029+0.158 | 2.637+0.143 | 1.237+0.191 | | 2.579+0.158 |
| **Third-**  **order root** | **NF** | 0.540+0.078 | 1.054+0.173 | 0.692+0.126 | | 1.078+0.142 |
|  | **FC** | 0.575+0.055 | 1.400+0.049 | 0.842+0.015 | | 1.533+0.050 |
|  | **FNC** | 1.515+0.084 | 1.977+0.119 | 1.026+0.149 | | 1.457+0.128 |
|  | **F** | 0.711+0.084 | 1.892+0.143 | 0.674+0.060 | | 1.790+0.072 |
| **Fourth-**  **order root** | **NF** | 0.538+0.111 | 0.927+0.036 | 0.427+0.053 | | 0.851+0.089 |
|  | **FC** | 0.673+0.017 | 1.365+0.190 | 0.895+0.169 | | 1.153+0.030 |
|  | **FNC** | 1.491+0.153 | 1.514+0.014 | 1.161+0.038 | | 1.379+0.162 |
|  | **F** | 1.185+0.246 | 1.844+0.263 | 0.810+0.044 | | 1.903+0.035 |
