# Supplementary material for: Responses of nutrient capture and fine root morphology of subalpine coniferous tree Picea asperata to nutrient heterogeneity and competition
Source: PLoS One. 2017 Nov 2;12(11):e0187496. doi: 10.1371/journal.pone.0187496 (PMC5667764; doi:10.1371/journal.pone.0187496)
Supplement: S5 Table — (DOCX) [file pone.0187496.s005.docx]

**S5 Table. The concentrations of K and P in roots of different branch order affected by the competition and its interaction with nutrients heterogeneity in the competitive and non-competitive compartments (means + SE, n=8).**

|  | **Treatments** | **K**  **(mg/g)** | **P**  **(mg/g)** |
| --- | --- | --- | --- |
| **Stem** | **SNF** | 2.440+0.162 | 1.121+0.086 |
|  | **SHF** | 2.513+0.064 | 1.390+0.016 |
|  | **SF** | 2.673+0.042 | 1.504+0.020 |
|  | **NF** | 2.56+0.128 | 0.881+0.035 |
|  | **FC** | 2.635+0.051 | 1.067+0.027 |
|  | **FNC** | 2.469+0.118 | 1.168+0.026 |
|  | **F** | 3.063+0.195 | 1.678+0.045 |
| **Branch** | **SNF** | 3.819+0.720 | 1.261+0.249 |
|  | **SHF** | 4.734+0.069 | 1.953+0.026 |
|  | **SF** | 5.176+0.266 | 2.085+0.087 |
|  | **NF** | 4.316+0.286 | 1.041+0.090 |
|  | **FC** | 4.473+0.103 | 1.458+0.032 |
|  | **FNC** | 4.435+0.094 | 1.523+0.043 |
|  | **F** | 4.835+0.321 | 1.761+0.133 |
| **Leaf** | **SNF** | 5.802+0.648 | 1.996+0.173 |
|  | **SHF** | 5.471+0.323 | 2.467+0.049 |
|  | **SF** | 5.478+0.136 | 2.612+0.047 |
|  | **NF** | 6.173+0.306 | 2.045+0.075 |
|  | **FC** | 6.744+0.357 | 2.376+0.074 |
|  | **FNC** | 6.401+0.119 | 2.498+0.046 |
|  | **F** | 6.413+0.344 | 2.716+0.197 |
